# Primary Culture of Hippocampal Neurons from P0 Newborn Rats

**DOI:** 10.3791/895

**Published:** 2008-09-29

**Authors:** Joseph Nunez

**Affiliations:** Department of Psychology, Michigan State University

## Abstract

The physiological properties of hippocampal neurons are commonly investigated, especially because of the involvement of the hippocampus in learning and memory. Primary hippocampal cell culturing allows neuroscientists to examine the activity and properties of neurons at the individual cell and single synapse level. In this video, we will demonstrate how to isolate and grow primary hippocampal cells from newborn rats. The hippocampus may be isolated from each newborn animal in as short as 2 to 3 minutes, and the cultures can be maintained for up to two weeks. We will also briefly demonstrate how to use these hippocampal neurons for ratiometric calcium imaging. While this protocol describes the process for the hippocampus, with little to no modification, it can be applied to other regions of the brain.

**Figure:**
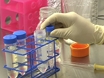


## Protocol

### Prior to hippocampal isolation

Before beginning the hippocampal isolation, make sure that all the tools are sterile. Spray down the culture hood with 70% ethanol, and place the tools inside the hood. You will need 6- and 10-cm Petri dishes, sterile poly-L coated glass coverslips, pipettors and tips, disposable pipettes, and an electric pipettor. From this point on, remember to use correct sterile technique.

Turn on the water bath and make sure that it is heated up to 37°C.
The following solutions that are stored at 4°C will be needed:
  Modified Eagle’s Medium (MEM)
  Neurobasal
  Hank’s buffered saline solution (HBSS)
  Borate buffer solution
  Sodium pyruvate solution
  Sterile-filtered 20% glucose solution in distilled water
  Make sure to spray all the bottles down with 70% ethanol before placing them in the hood.
Take these frozen solutions out of the freezer and place them in a heated water bath:
  A 5 ml aliquot of horse serum
  A 1 ml aliquot of B-27 supplement
  A 1 ml aliquot of 100X antibiotic (penicillin plus streptomycin)
  And a 0.5 ml aliquot of L-glutamine
After the solutions have thawed, take them out of the water bath, spray them down with 70% ethanol, and place them in the hood. Now the plating and Neurobasal medium can be prepared.
After the solutions are prepared, cap the containers tightly and place them in the 37°C bath to warm up while performing the hippocampal isolation.
Also, take an aliquot of the protease trypsin (2.5%) out of the freezer and place it in the water bath. Trypsin will digest the dissected hippocampus, which will be isolated in the next step.
Take a 15ml conical tube, fill it with HBSS, and label it with the treatment group. It is here that isolated hippocampi will be collected in the next step.

### Hippocampal Isolation

To begin the hippocampal isolation, make sure newborn pups are clean and have had their milk bands, placentas, and umbilical cords removed by their mother.
Place the pups in a Petri dish, spray with 70% ethanol to clean, and place them in the hood. Animals are euthanized immediately before culturing.
For further sterilization take one pup from the dish, dip the pup into 70% ethanol, and then into two washes of sterile HBSS.
Remove the head from the body with scissors. Using the same scissors, cut through the skin and the skull.
Using a pair of fine tweezers, peel the skull away from the brain and place the brain into a small Petri dish that contains a small amount of sterile HBSS.
Peel back the cerebral hemispheres. The hippocampus is a small, seahorse-shaped structure in the medial temporal lobe.
Remove the hippocampus and place into 3 ml of HBSS in a 15 ml tube. Repeat these steps with each pup, and place each isolated hippocampus into the 15 ml tube. Now, it is time to dissociate the tissue to single cells.

### Hippocampal cell dissociation

After all hippocampi have been isolated, fill the 15 ml tube to 4.5 ml with HBSS.
Remove the trypsin from the water bath, spray with ethanol, and place in the hood. Add 0.5 ml of trypsin to the tube, and incubate for 15 minutes at 37°C.
In the hood, remove the HBSS/trypsin solution from the tube with a sterile pipette, being careful not to disturb the hippocampi that have settled to the bottom of the tube. Add 5 ml of HBSS to the tube and swirl gently. Incubate at 37°C for 5 minutes.
Repeat this step twice, removing the old HBSS and replacing with fresh solution.
Take an aliquot of DNAse I out of the freezer. Add 0.5 ml of DNase to the hippocampi in 4.5 ml HBSS. The DNAse is added to promote enzyme inactivation.
Triturate the solution (or pipette up and down) until it is homogenous. Be careful not to introduce bubbles into the homogenate.
Determine cell viability with the trypan blue exclusion method.
Take a 10 cm Petri dish containing 6 to 8 coverslips, and add 10 ml of plating medium. Pipette the desired number of cells to the dish containing the coverslips. Swirl gently to disperse the cells, and make sure the coverslips do not overlap.
Allow the cells to attach for 2-4 hours in a humidified 37°C incubator with 5% CO_2_. After confirming that the cells are viable and have attached, transfer the coverslips to individual dishes containing Neurobasal medium. Place these dishes back into the incubator.
Once a week, replace one-third of the medium with fresh Neurobasal medium and experimental treatments, as needed. Now, the functional properties of these cells in culture are ready to be studied.

### Fura-2 Calcium Imaging of Hippocampal Neurons

After cultured hippocampal neurons have been *in vitro* for 48 hours (but not earlier), calcium imaging experiments can begin. At this point, these neurons should have begun to extend processes. The optimal age range for calcium imaging is from day *in vitro* 3 to 7.
Load cultures with Fura-2 AM for 30 minutes at 37°C, after which they can be imaged under a 20X objective using a Xenon arc lamp to excite the calcium indicator dye and a 512 bit ccd camera, which samples every 150ms.

## Discussion

This protocol was developed as a modification of the seminal work of Gary Banker and Kim Goslin ^1^. This book is an essential resource for anyone interested in culturing cells -- not only the neuron-enriched cultures described in the current protocol.

The three most critical factors in cell culturing are: sterility, speed, the choice of medium used.

**Sterility** - a laminar flow/sterile hood, aseptic conditions, and a sterile incubator are required. Contamination at any step is detrimental not only to the current experiment you are working on, but also the subsequent work that may have been planned. After an incubator becomes contaminated, it may take weeks (or a month) to get it cleaned and sterile. Contamination, while not immediately evident at a visible level, will definitely affect cell viability and cell physiology. Sterility is imperative.

**Speed** - the health of cultured cells is strongly tied to the amount of time the cells are not in an atmosphere and medium controlled environment. Therefore, it is essential that the amount of time required to remove cells from the animal until the cells are in the plating medium is at a minimum. Because numerous steps cannot be altered (time in incubating in trypsin or being washed), what can be altered is the amount of time required to remove the cells from the brain. Practice the dissection often. The amount of time required for this step needs to be reproducible.

**Choice of medium** - there are numerous proprietary mediums, one of which is Neurobasal. Prior to the days of using Neurobasal, glial conditioned medium (which needed to be made separately - this is discussed in the book by Banker and Goslin ^1^) was required. Regardless, it is important that you know what is in the medium that is being used. Along with Neurobasal, a supplement such as B-27 is often used. Make sure that the constituents of these medium and supplements are known, as they may affect the properties of the cells. It is standard practice in my lab to use serum free/charcoal stripped/phenol red free medium   to avoid steroid hormones (given that a large portion of the work performed in my lab investigates the actions of steroid hormones). This concept -- *know what is in the solutions you are using* -- is critical for all steps in cell culturing.
